# Campylobacter beyond the gut: A case series of *Campylobacter jejuni* bacteraemia with atypical presentations

**DOI:** 10.1177/00494755261466366

**Published:** 2026-07-06

**Authors:** Anna Rajan Thomas, Kavya Samhitha, Nivedha Srinivasan, Anurag Shetty, Shivraj Padiyar U, Sathish B Rao

**Affiliations:** 1 Senior Resident, Department of Microbiology, 76798 Kasturba Medical College Mangalore, Manipal Academy of Higher Education, Manipal, India; 2 Junior Resident, Department of General Medicine, 76798 Kasturba Medical College Mangalore, Manipal Academy of Higher Education, Manipal, India; 3 Associate Professor, Department of Gastroenterology, 76798 Kasturba Medical College Mangalore, Manipal Academy of Higher Education, Manipal, India; 4 Professor, Department of Clinical Immunology and Rheumatology, Kasturba Medical College Mangalore, Manipal Academy of Higher Education, Manipal, India; 5 Head and Professor, Department of General Medicine, Kasturba Medical College Mangalore, Manipal Academy of Higher Education, Manipal, India

**Keywords:** Campylobacter jejuni, bacteraemia, atypical presentation, immunocompromised host, diagnostic challenge

## Abstract

*Campylobacter jejuni* is a common cause of gastro-enteritis, whereas bacteraemia is an infrequent and under-recognized manifestation. Initial standard culture conditions failed to yield growth, indicating possible diagnostic delays. Identification was achieved by incubating the culture plates in microaerophilic conditions and rapid identification by MALDI-TOF MS (Matrix-Assisted Laser Desorption/Ionization Time-of-Flight Mass Spectrometry).

## Introduction

*Campylobacter jejuni is responsible for entero-invasive diarrhoea.* Bacteraemia is rare, and when it occurs, *Campylobacter fetus* is more frequently isolated.^1^ Unusual manifestations and diagnostic challenges associated with these infections may lead to potential delays in diagnosis and treatment.

## Case series

### Case 1

A male patient in his mid-sixties presented with complaints of breathlessness and jaundice for five days. He was undergoing treatment for tuberculous ascites on ethambutol and levofloxacin (modified antitubercular treatment) for two months, had chronic kidney disease on maintenance haemodialysis, diabetes mellitus with diabetic retinopathy, and systemic hypertension on medication for three years. In his past medical history, there was history of repeated admissions for abdominal distension requiring multiple ascitic taps, but no history of foreign food intake nor consumption of uncooked meat. He was normotensive (140/90 mmHg) and had mild to moderate ascites. Initial laboratory evaluation demonstrated anaemia, deranged liver function tests, and markedly elevated serum creatinine levels (0.046 mmol/L); in view of the recent initiation of antitubercular therapy, drug-induced hepatitis was initially suspected.

**Figure 1.**
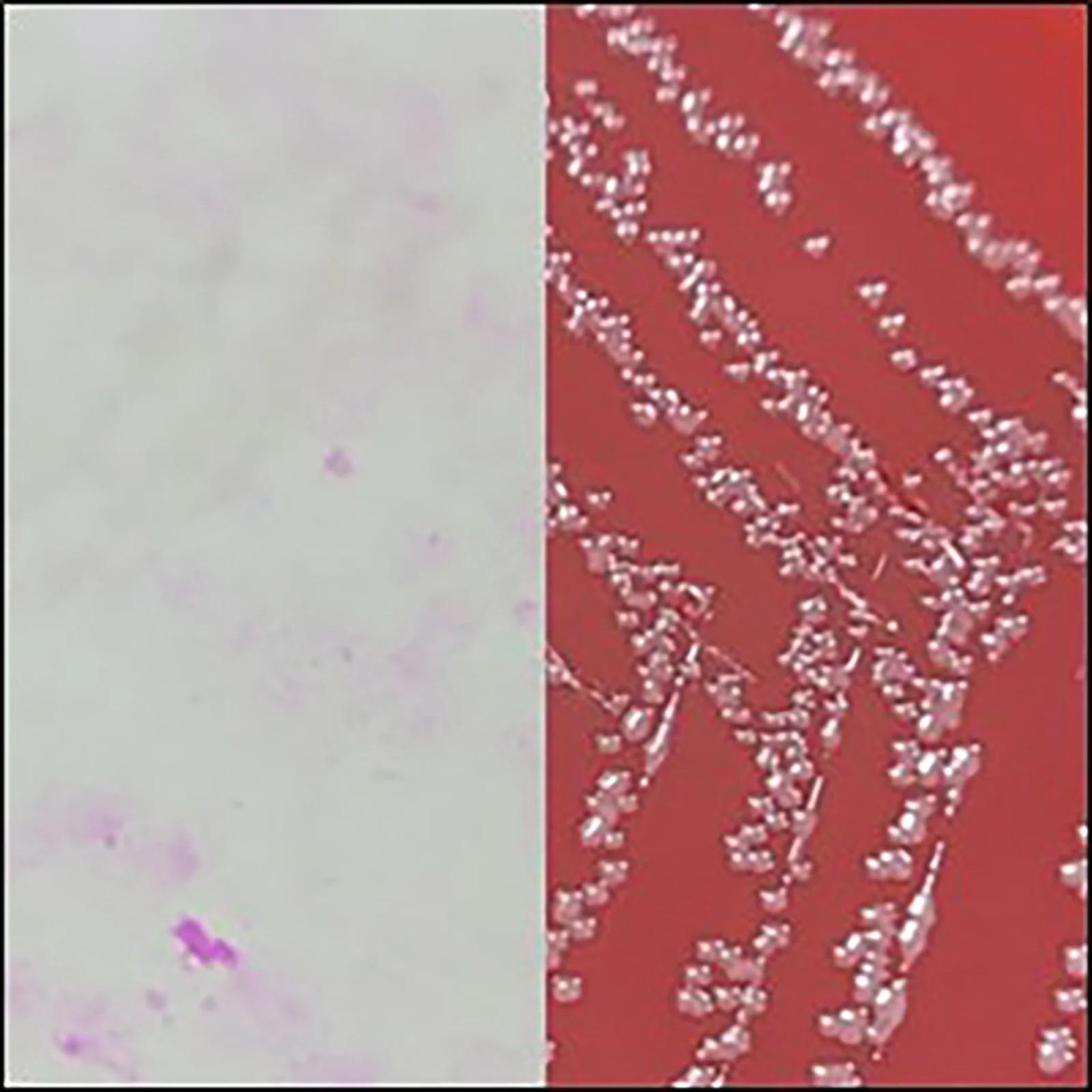
(A) Gram-stained picture: curved spiral, S-shaped or gull-winged, or long spiral gram-negative bacilli. (B) *C. jejuni* growth on blood agar plate.

Paired automated blood cultures (BD BACTEC^TM^) were sent from his arteriovenous fistula site, which flagged positive within 48h. Although the initial Gram stain was reported as inconclusive, careful re-evaluation of the smear revealed thin, fragile, Gram-negative bacilli (Fig. 1). No growth was observed on routine culture the following day. On suspicion of a fastidious organism, plates were placed in a 5% CO_2_ incubator for 48h, after which growth was observed on the blood and chocolate agar plates (Fig. 1). Identification from the colonies by Matrix-Assisted Laser Desorption/Ionization Time-of-Flight Mass Spectrometry (MALDI-TOF MS) (Biomerieux^TM^) revealed *Campylobacter jejuni* subspecies *jejuni*. Cefoperazone-sulbactam which was started empirically was continued as the patient's clinical condition improved. At discharge, azithromycin 500 mg od for three days was prescribed.

### Case 2

A female in her early thirties with recently diagnosed IgA vasculitis (skin and renal involvement) on oral prednisolone 50 mg od and mycophenolate presented with a day's history of severe, diffuse abdominal pain, predominantly in the left iliac region, vomiting, multiple episodes of large-volume rectal bleeding and generalised weakness, without fever or chills. A history of consumption of leftover salad was noted.

She was hypotensive (90/60 mmHg). Initial laboratory evaluation showed leucocytosis with neutrophilia, while inflammatory markers remained within normal limits. Piperacillin-tazobactam IV was started empirically. An abdominal CT scan suggested colitis, which was presumed to be autoimmune, and hydrocortisone injections at 50 mg tds was started. Stool culture was negative, but blood culture yielded a growth of Gram-negative, curved, S-shaped bacilli suspected to be *Campylobacter* species after 44h. Based on the previous case, the plates were promptly incubated under appropriate conditions and identified as *Campylobacter jejuni* by MALDI-TOF MS, presumed responsible for her colitis. Steroids were tapered to 0.5 mg/kg and mycophenolate was withheld. Azithromycin 500 mg od was administered for seven days. Repeated blood cultures were negative, and our patient could be discharged with no active complaints.

### Case 3

A male patient in his early seventies, with a 6-month history of decompensated chronic liver disease and known hypothyroidism on levothyroxine, presented with progressive abdominal distension, bilateral lower-limb oedema, fever and chills and grade-2 hepatic encephalopathy. On admission, he was haemodynamically stable. Respiratory examination revealed bilateral wheezes, and abdominal evaluation demonstrated gross ascites.

Initial laboratory tests showed normocytic normochromic anaemia (Hb 96 g/L), an elevated ESR of 71 mm/h, and deranged liver function tests with hypoalbuminemia (23 g/L). Ascitic fluid analysis revealed a high serum-ascites albumin gradient pattern, with negative adenosine deaminase and no evidence of spontaneous bacterial peritonitis. Routine stool examinations showed no abnormalities. *Campylobacter jejuni* was isolated from blood culture after 31h. Oral azithromycin 500 mg od was supplied for ten days, with standard anti-encephalopathy measures and supportive management for his chronic liver disease. He showed gradual clinical improvement, resolution of encephalopathy, a reduction in systemic symptoms, and was discharged in a stable condition.

## Discussion

*Campylobacter jejuni* bacteraemia is an uncommon manifestation (seen in <1% of patients) and is probably under-recognized in clinical practice.^1^ It occurs predominantly in males with chronic liver disease and multiple comorbidity, including chronic liver or renal disease, and immuno-suppression,^2–4^ with fever and abdominal pain being common, and diarrhoea seen in 17%.^5^

In our cohort, gastrointestinal manifestations were variable; two patients did not experience diarrhoea, whilst the second case had acute gastrointestinal symptoms. Here radiological features of colitis were initially attributed to IgA vasculitis, but subsequent identification of *C. jejuni* and her clinical response to therapy suggested an infectious contribution. Overall, this highlights the ability of *Campylobacter jejuni* to cause bacteraemia in atypical presentations, leading to delays in management.^6–9^ From a diagnostic perspective, *Campylobacter* should be considered when slender, curved Gram-negative bacilli are seen on Gram stain and appropriate processing under micro-aerophilic conditions should then be pursued. Although most gastro-intestinal infections are self-limiting, bacteraemia in high-risk patients warrants prompt antimicrobial therapy. All our patients responded well to macrolides; however, emerging resistance may limit empirical treatment options.^10^
